# Hormetic Effect of Chronic Hypergravity in a Mouse Model of Allergic Asthma and Rhinitis

**DOI:** 10.1038/srep27260

**Published:** 2016-06-02

**Authors:** Tae Young Jang, Ah-Yeoun Jung, Young Hyo Kim

**Affiliations:** 1 Department of Otorhinolaryngology, Head and Neck Surgery, Inha University College of Medicine, Incheon, Republic of Korea

## Abstract

We aimed to evaluate the effect of chronic hypergravity in a mouse model of allergic asthma and rhinitis. Forty BALB/c mice were divided as: group A (n = 10, control) sensitized and challenged with saline, group B (n = 10, asthma) challenged by intraperitoneal and intranasal ovalbumin (OVA) to induce allergic asthma and rhinitis, and groups C (n = 10, asthma/rotatory control) and D (n = 10, asthma/hypergravity) exposed to 4 weeks of rotation with normogravity (1G) or hypergravity (5G) during induction of asthma/rhinitis. Group D showed significantly decreased eosinophils, neutrophils, and lymphocytes in their BAL fluid compared with groups B and C (*p* < 0.05). In real-time polymerase chain reaction using lung homogenate, the expression of *IL-1β* was significantly upregulated (*p* < 0.001) and *IL-4* and *IL-10* significantly downregulated (*p* < 0.05) in group D. Infiltration of inflammatory cells into lung parenchyma and turbinate, and the thickness of respiratory epithelium was significantly reduced in group D (*p* < 0.05). The expression of *Bcl-2* and *heme oxygenase-1* were significantly downregulated, *Bax* and *extracellular dismutase* significantly upregulated in Group D. Therefore, chronic hypergravity could have a hormetic effect for allergic asthma and rhinitis via regulation of genes involved in antioxidative and proapoptotic pathways. It is possible that we could use hypergravity machinery for treating allergic respiratory disorders.

Space physiology, which deals with physiologic changes in space, is an emerging field of research as the need for space exploration increases. For more successful performance of several missions in space, thorough understanding of the physiologic changes associated with spaceflight is quite mandatory. The environmental challenges when an organism is exposed to spaceflight include phychological stress, radiation, and abrupt change in inertial condition (such as hyper- or micro-gravity)^1^,^2^.

Due to economic burden and limited opportunity, it is extremely difficult to perform experiments in real space. Therefore, a number of ground-based models have been developed to simulate space environment. One of the most widely accepted model for simulating hypergravity is the centrifugal device. By using the centrifugal force due to the rotation, we could expose cultured cells or experimental animals to higher gravity than 1 G^3^,^4^,^5^,^6^.

The immune system is one of the most affected biological systems when exposed to space stimuli. In fact, immune dysfunction has been suggested as a major health problem during long-term space mission^7^. However, researches on the impact of hypergravity on the immune system is still limited. Some studies have evaluated changes in the mitogen-induced proliferation of lymphocytes, titers of several cytokines, and subpopulations of lymphocytes^1^,^8^,^9^,^10^,^11^,^12^,^13^,^14^,^15^,^16^.

Generally, space stimuli are thought to be noxious and harmful. However, in certain circumstances, hypergravity could have a beneficial effect on the human body. Ling *et al*. suggested that when rat bone marrow mesenchymal stem cells were exposed to hypergravity and 5-azacytidine at the same time, their differentiation into cardiomyocytes was significantly improved^4^. Exposure to hypergravity could also upregulate expression of osteopontin in osteoblasts^5^, and enhance differentiation of neuronal cells^6^. However, to the best of our knowledge, studies on the effects on allergic immunity are scarce. Furthermore, according to our review of the literature, no study has been conducted on the effect of hypergravity on animals with allergic disorders. When we exposed mice with allergic asthma and rhinitis to chronic hypergravity (5 G, 4 weeks), we could observe significant improvement in their clinical course of allergic airway inflammation. To establish the physiologic mechanisms underlying this clinical improvement, we evaluated (1) serum total and ovalbumin (OVA)-specific IgE, (2) number of inflammatory cells in bronchoalveolar lavage (BAL) fluid, (3) expression of genes for *IL-1β, IL-4, IL-5, IL-6, IL-10,* and *interferon (IFN*)*-γ* in lung homogenate, and (4) histopathologic findings of lung and nasal cavity. We also performed real-time polymerase chain reaction (PCR) for *Bcl-2, Bax, caspase-3, heme oxygenase (HO*)*-1* and *extracellular superoxide dismutase (EC-SOD*).

## Results

### Rotatory Stimulus and Chronic Hypergravity Caused a Significant Decrease of Serum Total IgE But Not OVA-specific IgE

**Figure 1:**
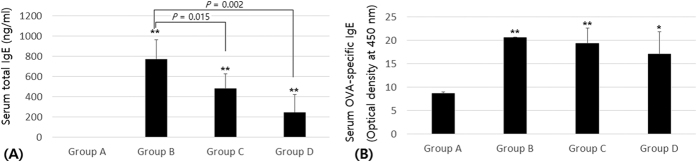
(A) Serum total IgE. Compared with group A, group B had significantly elevated serum total IgE. After 4 weeks of continuous exposure to 1G or 5G gravity, the mice in groups C and D had significantly decreased serum total IgE (*p* < 0.05). **(B**) Serum ovalbumin (OVA)-specific IgE. Compared with group A, groups B and C had significantly elevated OVA-specific IgE (*p* < 0.01). After 4 weeks of 5G hypergravity, group D had no significant decrease of OVA-specific IgE compared with groups B or C (*p* > 0.05). Group A is the control group, group B is the asthma group, group C is the asthma/rotatory control group, and group D is the asthma/hypergravity group. *Significant difference from group A, *p* < 0.05; **significant difference from group A, *p* < 0.01 (Kruskal-Wallis and Mann-Whitney U tests).

Compared with group A (control group), group B (asthma group) had a significantly elevated serum total IgE level (*p* < 0.01). After 4 weeks of continuous exposure to 1G or 5G gravity, the mice in group C (asthma/rotatory control group) and group D (asthma/hypergravity group) had significantly decreased serum total IgE levels (*p* < 0.05, Fig. 1A).

Compared with group A, groups B and C had significantly elevated OVA-specific IgE levels (*p* < 0.01). After 4 weeks of continuous exposure to 5G hypergravity, group D had no significant decrease of OVA-specific IgE level compared with group B or C (*p* > 0.05, Fig. 1B).

### Decrease of Inflammatory Cells in BAL Fluid after Exposure to 4 Weeks of Hypergravity

**Figure 2:**
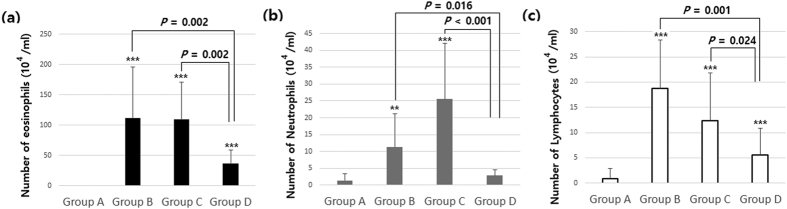
The number of (**a**) eosinophils, (**b**) neutrophils, and (**c**) lymphocytes in bronchoalveolar lavage (BAL) fluid. Compared with group A, the number of eosinophils, neutrophils, and lymphocytes in BAL fluid was significantly increased in groups B and C (*p* < 0.05). After exposure to 4 weeks of hypergravity, group D showed significant decreases of all these inflammatory cells in BAL fluid compared with groups B and C (*p* < 0.05). Group A is the control group, group B is the asthma group, group C is the asthma/rotatory control group, and group D is the asthma/hypergravity group. **Significant difference from group A, *p* < 0.01; ***significant difference from group A, *p* < 0.001 (Kruskal-Wallis and Mann-Whitney U tests).

Compared with group A, the number of inflammatory cells such as eosinophils, neutrophils, and lymphocytes in BAL fluid was significantly increased in groups B and C (*p* < 0.05). After exposure to 4 weeks of hypergravity, group D showed a significant decrease of all these inflammatory cells in BAL fluid compared with groups B and C (*p* < 0.05). Regarding neutrophils, especially, there was no significant difference between groups A and D (*p* > 0.05, Fig. 2).

### Chronic Hypergravity Caused upregulation of IL-1β and downregulation of IL-4 and IL-10 in Lung Homogenate

**Figure 3:**
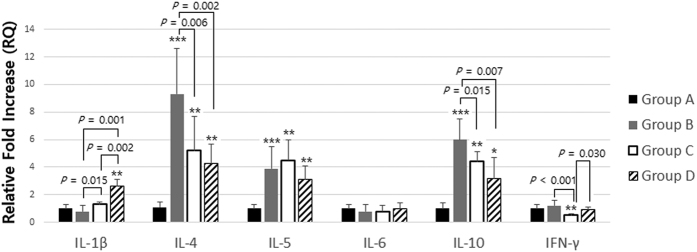
Gene expression for cytokines in lung homogenate. Group D had significant upregulation for *IL-1β* compared with group B or C (*p* < 0.01). For *IL-4* and *IL-10*, groups C and D had significant downregulation compared with group B after 4 weeks of rotatory normogravity stimulus or hypergravity (*p* < 0.05). Although the expression of *IL-5* showed some tendency to be decreased in group D, there was no statistically significant difference (*p* > 0.05). For *IFN-γ*, group C had significant downregulation (group B versus C, *p* < 0.001), which was recovered to a normal level in group D (group C versus D, *p* = 0.030). Group A is the control group, group B is the asthma group, group C is the asthma/rotatory control group, and group D is the asthma/hypergravity group. *Significant difference from group A, *p* < 0.05; **significant difference from group A, *p* < 0.01; ***significant difference from group A, *p* < 0.001 (Kruskal-Wallis and Mann-Whitney U tests). RQ, relative quantification.

Performing real-time PCR analysis of lung homogenates, we found that group D had significant upregulation for *IL-1β* compared to groups B or C (*p* < 0.01). For *IL-4* and *IL-10*, groups C and D had significant downregulation compared to group B after 4 weeks of rotatory normogravity or hypergravity stimuli (*p* < 0.05). Although the expression of IL-5 showed some tendency to decrease in group D, there was no statistically significant difference (*p* > 0.05). For IFN-γ expression, group C had significant downregulation (group B versus C, *p* < 0.001), which was recovered to normal level in group D (group C versus D, *p* = 0.030) (Fig. 3).

### Histopathologic Change Related to Exposure to Long-Term Hypergravity

**Figure 4:**
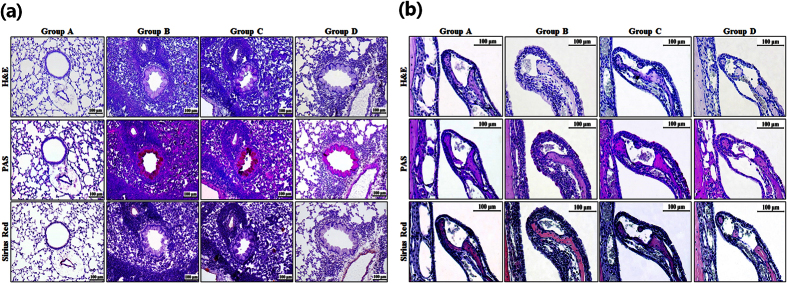
Histologic change of (**a**) lung and (**b**) nasal cavity after exposure to long-term hypergravity. Compared with group A, mice in groups B and C had more infiltration of inflammatory cells into pulmonary parenchyma. After exposure to hypergravity, mice in group D had significantly less infiltration of inflammatory cells. Histopathologic examination of the nasal cavity also revealed a significant tendency of less infiltration of inflammatory cells in the lamina propria in the turbinate in group D compared with groups B and C. Group A is the control group, group B is the asthma group, group C is the asthma/rotatory control group, and group D is the asthma/hypergravity group. The upper rows show hematoxylin and eosin (H&E) staining, the middle rows show periodic acid–Schiff (PAS) staining, and the lower rows show Sirius Red staining at ×200 magnification for lung tissue and ×400 magnification for nasal cavity. Scale bar: 100 μm.

Compared with mice in group A, those in groups B and C had more infiltration of inflammatory cells into pulmonary parenchyma. After exposure to 4 weeks of 5G hypergravity, mice in group D had significantly less infiltration of inflammatory cells. Histopathologic examination of the nasal cavity also revealed a significantly less infiltration of inflammatory cells in the lamina propria in the turbinate in group D compared with groups B and C (Fig. 4).

**Figure 5:**
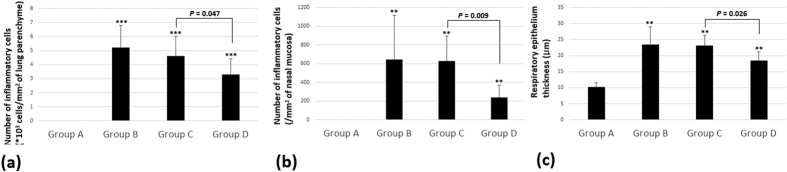
The number of inflammatory cells in (**a**) 1 mm^2^ of lung parenchyma and (**b**) 1 mm^2^ of lamina propria of nasal mucosa. (**c**) The thickness of respiratory epithelium. Mice in group D had a significantly decreased number of inflammatory cells that had infiltrated into pulmonary parenchyma and into the nasal mucosa compared with groups B and C. The thickness of the respiratory epithelium was significantly decreased in group D after exposure to chronic hypergravity. Group A is the control group, group B is the asthma group, group C is the asthma/rotatory control group, and group D is the asthma/hypergravity group. **Significant difference from group A, *p* < 0.01; ***significant difference from group A, *p* < 0.001 (Kruskal-Wallis and Mann-Whitney U tests).

In quantitative analysis, mice in group D had a significantly decreased number of inflammatory cells that had infiltrated into pulmonary parenchyma and into the nasal mucosa compared with groups B and C. The thickness of the respiratory epithelium was also significantly decreased in group D after exposure to chronic hypergravity (*p* < 0.05, Fig. 5).

### Change of Gene Expression Related to Apoptotic and Antioxidant Enzymes

Compared with group A, mice in group B had significantly upregulated gene expression for *Bcl-2 (p* < 0.05), *Bax (p* < 0.01), *caspase-3 (p* < 0.01), and *HO-1 (p* < 0.05) and significantly downregulated expression for *EC-SOD (p* < 0.05). After 4 weeks of hypergravity, group D had significant downregulation of *Bcl-2* and significant upregulation of *Bax* compared with group B (*p* = 0.009 for *Bcl-2*; *p* = 0.026 for *Bax*). However, there was no significant difference in the expression of *caspase-3* among groups B to D (*p* > 0.05).

**Figure 6:**
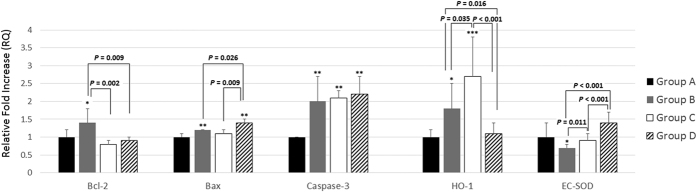
Gene expression related to apoptotic and antioxidant enzymes. Compared with group A, the mice in group B had significantly upregulated gene expression for *Bcl-2 (p* < 0.05), *Bax (p* < 0.01), *caspase-3 (p* < 0.01), and *HO-1 (p* < 0.05) and significantly downregulated expression for *EC-SOD (p* < 0.05). After 4 weeks of hypergravity, group D had significant downregulation of *Bcl-2* and significant upregulation of *Bax* compared with group B (*p* = 0.009 for *Bcl-2*; *p* = 0.026 for *Bax*). However, there was no significant difference in the expression of *caspase-3* among groups B to D (*p* > 0.05). Group D also showed significant suppression of *HO-1*. For *EC-SOD*, mice in groups C and D showed significant recovery of *EC-SOD* compared with group B (group B versus C, *p* = 0.011; group C versus D, *p* < 0.001). Group A is the control group, group B is the asthma group, group C is the asthma/rotatory control group, and group D is the asthma/hypergravity group. *Significant difference from group A, *p* < 0.05; **significant difference from group A, *p* < 0.01 (Kruskal-Wallis and Mann-Whitney U tests). RQ, relative quantification.

After chronic exposure to hypergravity, mice in group D also showed significant suppression of *HO-1*. For *EC-SOD*, finally, mice in groups C and D showed significant recovery of *EC-SOD* compared with group B (group B versus C, *p* = 0.011; group C versus D, *p* < 0.001) (Fig. 6).

## Discussion

There have been reports about the hormetic effects of chronic hypergravity in living organisms^3^,^4^,^5^,^6^. Minois suggested that when *D. melanogaster* was exposed to 2 weeks of hypergravity (3 to 5G), it lived longer (in other words, hormetic effects on its longevity)^17^. To the best of our knowledge, our study is the first to demonstrate the hormetic immune effect in animals with allergic disorder.

Only few studies have been conducted on the effect of hypergravity on the humoral immune system. Guéguinou and colleagues evaluated changes in serum IgG, IgA, and IgM after chronic exposure to 3 weeks of hypergravity up to 3G^18^. In our study, group C (asthma/rotatory control) and group D (asthma/hypergravity group) showed significant decreases of serum total IgE compared with group B (asthma/stationary control). As group C showed a significant decrease of serum total IgE with just rotatory stimulus (but without hypergravity), the decrease in IgE might be the result of a stress response to rotatory stimuli. Although no results showed a change in Ig levels after rotatory stimulus, Guéguinou and colleagues reported that serum titers of IgG were significantly increased after chronic exposure to 2G of hypergravity^18^. The serum titers of OVA-specific IgE in group D showed no significant difference from those in groups B and C. These results are in accordance with previous research. Voss and colleagues suggested that normal individuals showed no significant change in their humoral immunity after short-term spaceflight^19^. However, no studies have investigated the effect of chronic hypergravity in animals with allergic disorders. In our previous study, mice with allergic asthma showed no significant change in serum OVA-specific IgE after exposure to short-term hypergravity (10G for 4 hours)^20^. Therefore, we could suggest that changes in the allergic response in animals with allergic disorders may be unrelated to IgE-related mechanisms.

According to a previous study, the number of neutrophils and lymphocytes in serum was significantly reduced after exposure to 3 weeks of hypergravity up to 3G^21^. However, no study has evaluated the change of inflammatory cells in BAL fluid. In our study, the number of all inflammatory cells including eosinophils, neutrophils, and lymphocytes was significantly decreased after long-term exposure to hypergravity as shown in group D. We decided to identify the physiologic mechanism underlying this hormetic immune response in experimental animals.

After exposure to short-term hypergravity, serum titers of IL-1β showed no significant change^20^. On the other hand, Liu and colleagues reported that IL-1β titers in rat brain were significantly increased after short-term exposure to hypergravity of 14G^22^. In this study, the expression of *IL-1β* in lung homogenate was significantly increased, but that of *IL-4* and *IL-10* was significantly decreased after exposure to chronic hypergravity in allergic mice. Although not statistically significant, the expression of IL-5 also showed some tendency to decrease in group D. Therefore, we could postulate that the shift from a Th_2_ to a Th_1_ response (increased Th_1_ cytokines and decreased Th_2_ cytokines) could be partly responsible for the reduced allergic immune response. Regarding IFN-γ, Pecaut and colleagues suggested that the serum concentration was significantly decreased during a 3-week period of 3G hypergravity^3^. However, as they used stationary control animals (mice housed in stationary cages), the effect of rotatory stress could not be ruled out. In our study, group C showed significant down-regulation of IFN-γ after normogravity rotation. After long-term exposure to hypergravity, the expression of IFN-γ was restored to a normal level. As biological activity of IFN-γ is also related to promotion of Th_1_ differentiation and suppression of Th_2_ differentiation, the increase of IFN-γ after exposure to hypergravity could also be responsible for the shift from a Th_2_ to a Th_1_ response.

To confirm the effect of chronic hypergravity on the allergic inflammatory response in the lung and the nasal cavity, we performed a histopathologic examination. As there was a significant decrease in the number of inflammatory cells that had infiltrated into lung parenchyma and nasal mucosa, we could confirm histologically that chronic hypergravity could alleviate allergic asthma and rhinitis.

The mechanism of hormetic effect of chronic hypergravity still remains largely unveiled^17^. One of the hypotheses is that protection and repair mechanisms against the stress could be involved^17^. Because of increased metabolic rate due to increased weight, there could be more oxidative damage as a result. Therefore, activities of several anti-oxidant enzymes could be upregulated to protect an organism against this oxidative damage^17^. In order to investigate this hypothesis in our model of allergic inflammation, we evaluated the expression of several genes related with anti-oxidative and anti-apoptotic function.

The *Bcl-2* family comprises proteins involved in the intrinsic apoptotic pathway. Among them, *Bcl-2* is an antiapoptotic factor, and *Bax* is one of the proapoptotic factors. In our study, the expression of *Bcl-2* was significantly suppressed and that of *Bax* was significantly enhanced after exposure to chronic hypergravity. Suppression of antiapoptotic enzymes and enhancement of proapoptotic enzymes could be responsible for increased apoptosis (and decreased number) of inflammatory cells in the lung parenchyma and nasal cavity. A decrease of inflammatory cells, in turn, could be responsible for the decreased Th_2_ cytokines. Our results and suggestions are in agreement with those of previous studies. Lin and colleagues suggested that IL-21 treatment caused reduced allergic symptoms, increased apoptosis of Th_2_ lymphocytes, and a significant decrease of the antiapoptotic factor *Bcl-2* in allergic mice^23^. Zhao and colleagues reported that the expression of *Bcl-2* was significantly increased in patients with allergic rhinitis. They stated that the expression of *Bcl-2* was increased in CD4^+^ T lymphocytes and endothelial and epithelial cells^24^. On the other hand, the expression of *Bax* and *caspase-3* mRNA was significantly increased in allergic mice^25^. Therefore, a protective effect of chronic hypergravity against allergic inflammation could be related to the downregulation of antiapoptotic signals (*Bcl-2*) and upregulation of proapoptotic signals (*Bax*) in inflammatory cells such as eosinophils, neutrophils, and lymphocytes.

*HO-1*, a specific regulator of endogenous carbon monoxide, suppresses degranulation of mast cells and synthesis of Th_2_ cytokines. Therefore, it has a protective effect against allergic airway inflammation, airway hyperresponsiveness and hypersecretion of mucus^26^. Yu and colleagues suggested that OVA-sensitized guinea pigs showed significantly enhanced expression of the *HO-1* gene, which was significantly suppressed after dexamethasone treatment^27^. Kuribayashi and colleagues also suggested that suppression of *HO-1* activity by zinc protoporphyrin (a competitive *HO-1* inhibitor) resulted in inhibition of airway hyperresponsiveness and pulmonary eosinophilia^28^. Therefore, suppression of *HO-1* could be a promising potential target for antiallergic treatment^26^,^29^,^30^. In our study, it is probable that the suppression of *HO-1* in group D could be responsible for the antiallergic effect of chronic hypergravity.

*EC-SOD* is one of the most important enzymes, exerting its antioxidant activity by removing oxygen free radicals such as superoxide in the lung. In our study, expression of *EC-SOD* was significantly downregulated in allergic mice. Our results are in agreement with those of previous studies. Ono and colleagues suggested that SOD activity was significantly reduced in patients with chronic rhinosinusitis^31^. Kwon and colleagues also suggested that administration of *EC-SOD* alleviated the Th_2_ response in mice with allergic asthma. Also, *EC-SOD* knockout mice showed severer asthma^32^. Therefore, recovery of *EC-SOD* activity could be another possible pathway for the antiallergic effect of chronic hypergravity.

Someone could argue that 5G of hypergravity is so severe that this result could not be directly interpolated to human. However, we should keep in mind that sensitivity to gravitational force is so different from species to species. According to *Lee et al.*, 9G of hypergravity stimuli to mice equals 6.5 G to rats, and approximately matches to 2.7 G to humans^33^. According to them, 5 G of hypergravity used for this study would be equal to 1.3 to 1.4 G to human. As we need only modest speed of rotation and centrifugal force to induce 1.3 to 1.4 G of hypergravity, it would not cause much discomfort for the patient. Therefore, ‘hypergravity machinery’ for the new treatment strategy of allergic disorders would be relevant. Surely, we need to perform clinical study for human using bigger rotatory machinery to prove this possibility.

The importance of this study is that chronic hypergravity could have a positive effect on allergic asthma through activation of several genes involved in proapoptotic and antioxidant pathway. This is, to our best of knowledge, quite new finding and adds to our better understanding of the pathophysiology of allergic asthma^34^. Also, it is quite meaningful that hypergravity could possibly be considered as an additional, non-pharmacologic treatment in the management of allergic respiratory disorders.

Because of the small number of mice in each group, we had no choice but to perform nonparametric statistical analysis. Considering the large standard deviations for the results, it is necessary for us to perform further study with a larger population. Further study is also required to confirm the protein synthesis for all genes using Western blot analysis.

In conclusion, chronic hypergravity could have a beneficial effect in a mouse model of allergic asthma and rhinitis through regulation of genes involved in antioxidative and proapoptotic pathways.

## Methods

### Animals

Forty female BALB/c mice, 4 weeks old and free of murine-specific pathogens, were purchased from Orient Bio (Seongnam, Korea). They were raised in a controlled environment, with a regular 12-hour light/dark cycle and unrestricted access to OVA-free food and water. All mice used in this study were handled according to a protocol approved by the Institutional Animal Care and Use Committee (INHA 150309-351-2).

### Sensitization and Challenge

For induction of allergic asthma and rhinitis, mice were first sensitized with an intraperitoneal (i.p.) injection of 25 μg OVA (Sigma-Aldrich, St. Louis, MO, USA) and 1 mg aluminum hydroxide gel in sterile saline on days 0, 7, and 14. After systemic sensitization, mice were locally challenged by intranasal (i.n.) instillation with 500 μg OVA into their nostrils from days 21 to 27.

### Exposure to Hypergravity

We developed a gravitational force (G-force) simulator for hypergravity experiments, which has two rotatory arms (50 cm long). When the arms are rotated, an outward centrifugal force is exerted on the animal cage, which is suspended from the arms. When the arms rotate at a speed of 65 rpm, mice in the cage are exposed to 5G hypergravity. With a high-resolution video camera inside the cage, we could evaluate whether the mice could move freely and get access to food and water. In this experiment, mice were exposed to simulated hypergravity for 28 consecutive days, during the whole sensitization and challenge period. During this 28-day period, we stopped the G-force simulator once a day (for approximately 30 minutes), checked the vitality of the animals, facilitated food and water intake, and performed the i.p. sensitization or i.n. challenge.

**Figure 7:**
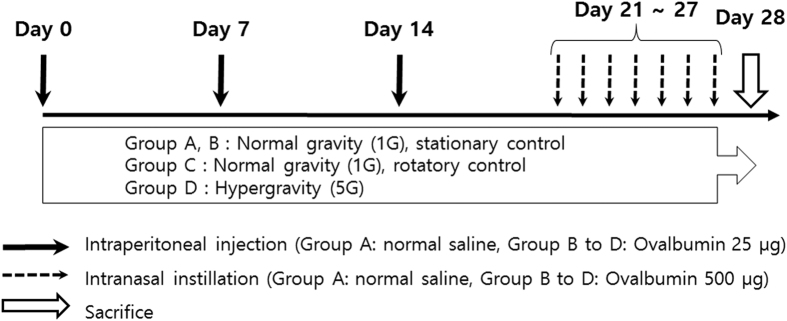
Study protocol for induction of asthma/rhinitis and exposure to gravity stimuli.

Mice in group A (n = 10, control group) received the i.p. and i.n. challenge with sterile saline only. Mice in group B (n = 10, asthma group) received the i.p. sensitization and i.n. challenge with OVA for induction of allergic asthma and rhinitis. Mice in groups A and B were bred without being exposed to any rotatory stimulus (stationary control). In group C (n = 10, asthma/rotatory control group), mice were exposed to a rotatory stimulus for 4 consecutive weeks as well as i.p. sensitization and i.n. challenge with OVA. However, with the centrifugal force so weak (lower rotational speed), animals in group C were exposed to normal gravity (1G, rotatory control). Finally, in group D (n = 10, asthma/hypergravity group), mice were exposed to continuous hypergravity of 5G for 28 days along with induction of allergic asthma and rhinitis (Fig. 7).

### Collecting Serum and Measuring Serum Total and OVA-specific IgE

Twenty-four hours after the last i.n. saline or OVA instillation, the G-force simulator was stopped and mice were immediately killed. We collected serum from the abdominal aorta using an aortic puncture technique. Whole blood was centrifuged at 4 °C for 30 minutes at 13,000 × *g*, and the supernatant was stored immediately at −80 °C. For analysis, the samples were diluted 1:100.

Serum levels of total IgE were measured using an enzyme-linked immunosorbent assay (ELISA) Total IgE was measured and compared with a mouse IgE standard (BD Pharmingen, San Diego, CA, USA). Serum titers for OVA-specific IgE were determined using an ELISA kit (BD Pharmingen). We used the plate-coated IgE-capture antibody with OVA 50 μg/mL, and analyzed the optical density at 450 nm in accordance with the protocol provided by manufacturer.

### Harvesting BAL Fluid and Differential Cell Counts in BAL Fluid

To harvest BAL fluid, we first cannulated the trachea using polyethylene tubing and then used a pulmonary lavage technique with sterile saline (approximately 3 mL). To determine the viability of cells and the total cell count, we used the trypan blue exclusion assay. Total cell numbers were determined in duplicates with a hemocytometer. Subsequently, a 100- to 200-μL aliquot was centrifuged in a Model 2 Cytospin cytocentrifuge (Shandon Scientific, Pittsburgh, PA, USA). Differential cell counts for eosinophils, neutrophils, and lymphocytes were determined from centrifuged preparations stained with the Diff-Quik stain kit (Sysmex Corp., Kobe, Japan) by counting 500 or more cells from each sample at a magnification of ×200 (oil immersion).

### Histopathologic examination

Tissue specimens of lung and nasal cavity were fixed in 4% paraformaldehyde solution for 24 hours. Lung tissues were washed with deionized water and then embedded in paraffin. Nasal tissues were also washed with deionized water and then immersed in EDTA solution for decalcification for 3 to 4 weeks. They were then embedded in paraffin in the same way. Tissue sections (3 μm thickness) were stained using hematoxylin and eosin solution (for qualitative evaluation of histopathologic change), periodic acid–Schiff solution (for mucus) and Sirius Red staining (for evaluation of eosinophilic infiltration). The number of eosinophils infiltrated into 1 mm^2^ of pulmonary parenchyma was counted in 20 random high-power fields (×200 magnification). The number of eosinophils infiltrated into 1 mm^2^ of lamina propria was counted for each sample in the T1 area (section immediately caudal to the upper incisor teeth) in 10 high-power fields (×400 magnification). The thickness of the respiratory epithelium was calculated using ImageJ software, calculating the number of pixels for the respiratory epithelial cells and dividing it by the total number of pixels in the entire lung field. Two impartial, blinded researchers performed the histopathologic examinations and counted the eosinophils in tissue specimens.

### Quantitative Real-time PCR

Whole-lung tissue from each mouse was frozen in liquid nitrogen immediately after harvest and homogenized in 1 mL TRIzol reagent (Invitrogen Life Technologies, Waltham, MA, USA). They were then stored at −20 °C until further analysis. Total RNA was isolated as recommended protocol by the manufacturer. Before complementary DNA synthesis, quantity of extracted total RNA was analyzed by NanoDrop spectrophotometer (Thermo Fisher Scientific Inc., Waltham, MA, USA). The integrity of the RNA was confirmed by electrophoresis.

After quantification of total RNA to 1 μg in each lung homogenate sample, we performed reverse transcription using the PrimeScript RT reagent kit (Takara Bio Inc., Shiga, Japan). Real-time PCR analysis was performed in duplicate using SYBR Green Master Mix ABI Prism in a PCR machine (7500 Real-Time PCR System; Applied Biosystems Inc., Carlsbad, CA, USA).

Primer annealing temperatures and number of cycling were as follows: 95 °C for 10 minutes, 40 cycles of 95 °C for 15 seconds and 60 °C for 1 minute. We identified the highlight Sequence Features of the target gene from the NCBI site and primers were designed using Primer 3.0 software (primer sequences are shown in Table 1, except for IL-5 and IL-10). Primers for IL-5 and IL-10 were purchased from QuantiTect Primer Assays (QIAGEN, Venlo, The Netherlands). To calculate the efficiency of amplification, the relative quantity of each target gene was normalized to the housekeeping gene *GAPDH*.

### Statistical Analysis

We used nonparametric tests such as the Kruskal-Wallis test and Mann-Whitney U test to compare the titers of total and OVA-specific IgE, the number of inflammatory cells in BAL fluid, the number of infiltrated eosinophils in pulmonary parenchyma and nasal cavity, and the degree of gene expression between groups. We considered a *p* value < 0.05 as statistically significant.

## Additional Information

**How to cite this article**: Jang, T. Y. *et al*. Hormetic Effect of Chronic Hypergravity in a Mouse Model of Allergic Asthma and Rhinitis. *Sci. Rep.* **6**, 27260; doi: 10.1038/srep27260 (2016).

## Figures and Tables

**Table 1:** Primer Sequences for Real-time Polymerase Chain Reaction.

**Gene**		**Primer Sequence**
*IL-1β*	Forward	5′-CTC GGC CAA GAC AGG TCG CTC-3′
	Reverse	5′-CCC CCA CAC GTT GAC AGC TAG G-3′
*IL-4*	Forward	5′-GGT CTC AAC CCC CAG CTA GT-3′
	Reverse	5′-TGT GAG GAC GTT TGG CAC AT-3′
*IL-6*	Forward	5′-GCC TTC TTG GGA CTG ATG CTG-3′
	Reverse	5′-GGA CTC TGG CTT TGT CTT TCT TGT-3′
*IFN-γ*	Forward	5′-AGG AAC TGG CAA AAG GAT GGT-3′
	Reverse	5′-GTT GCT GAT GGC CTG ATT GT-3′
*Bcl-2*	Forward	5′-GGA CTT GAA GTG CCA TTG GT-3′
	Reverse	5′-AGC CCC TCT GTG ACA GCT TA-3′
*Bax*	Forward	5′-GGA TGC GTC CAC CAA GAA GC-3′
	Reverse	5′-GGA GGA AGT CCA GTG TCC AGC C-3′
*Caspase-3*	Forward	5′-TGG GCC TGA AAT ACC AAG TC-3′
	Reverse	5′-AAA TGA CCC CTT CAT CAC CA-3′
*HO-1*	Forward	5′-CCT CAC TGG CAG GAA ATC ATC-3′
	Reverse	5′-CCT CGT GGA GAC GCT TTA CAT A-3′
*EC-SOD*	Forward	5′-GTG TCC CAA GAC AAT C-3′
	Reverse	5′-GTG CTA TGG GGA CAG G-3′
*GAPDH*	Forward	5′-GCA CAG TCA AGG CCG AGA AT-3′
	Reverse	5′-GCC TTC TCC ATG GTG GTG AA-3′
